# Mathematical Modeling of Intravascular Blood Coagulation under Wall Shear Stress

**DOI:** 10.1371/journal.pone.0134028

**Published:** 2015-07-29

**Authors:** Oleksii S. Rukhlenko, Olga A. Dudchenko, Ksenia E. Zlobina, Georgy Th. Guria

**Affiliations:** 1 National Research Center for Hematology, Moscow, Russia; 2 Moscow Institute of Physics and Technology, Dolgoprudny, Russia; 3 Cherkasy National University, Cherkasy, Ukraine; Gent University, BELGIUM

## Abstract

Increased shear stress such as observed at local stenosis may cause drastic changes in the permeability of the vessel wall to procoagulants and thus initiate intravascular blood coagulation. In this paper we suggest a mathematical model to investigate how shear stress-induced permeability influences the thrombogenic potential of atherosclerotic plaques. Numerical analysis of the model reveals the existence of two hydrodynamic thresholds for activation of blood coagulation in the system and unveils typical scenarios of thrombus formation. The dependence of blood coagulation development on the intensity of blood flow, as well as on geometrical parameters of atherosclerotic plaque is described. Relevant parametric diagrams are drawn. The results suggest a previously unrecognized role of relatively small plaques (resulting in less than 50% of the lumen area reduction) in atherothrombosis and have important implications for the existing stenting guidelines.

## 1 Introduction

The permeability of the vessel wall with respect to primary procoagulants plays an important role in the initiation of intravascular blood coagulation [1–8]. Within the context of the Virchow’s triad [9] variations in wall permeability contribute to vascular change and, alongside blood flow alterations [10, 11] and abnormalities of blood constituents, have been associated with numerous cases of clinical thrombosis [12].

Shear stress plays a pivotal role in controlling the barrier function of the vessel wall [8, 13–16]. In this paper we aim to investigate how increased shear stresses associated with stenosis—a local narrowing of the vessel wall—contribute to thrombogenicity of atherosclerotic plaques.

Up to now the analysis of blood coagulation under increased shear stress conditions has been primarily concerned with platelet activation—a distinct type of platelet response occurring in shear flows in which the shear rates exceed ∼ 5400*s* ^−1^ [17–19]. A number of reports however describe clotting induced by shear rates as low as 1000 *s* ^−1^ [5]—much lower than required for platelet activation. We speculate that some of these incidences can be explained by the shear stress-induced permeability of the vessel wall to procoagulants building up inside the atherosclerotic plaque.

To investigate this scenario we propose a mathematical model of blood coagulation in a vessel with a local stenosis and a source of procoagulants in the surrounding tissue. Importantly, the permeability of the vessel wall to these procoagulants is a function of wall shear stress.

Numerical analysis of the model reveals the existence of two thresholds in the hydrodynamic activation of blood coagulation in the system: one associated with the increase of wall permeability and another one linked to the washing out of procoagulants in the intensified blood flow. Additionally typical scenarios of intravascular clot formation are described and the relevant parametric diagrams of the blood liquid state stability are drawn.

The model also sheds some light onto the relationship between the thrombogenicity of the atherosclerotic plaque and it’s shape and severity. The results obtained are important for critical revision of the indications for stenting procedures currently used in the clinic.

## 2 Model description

Mathematical modeling of blood coagulation has been a subject of intensive research in the last three decades. Extended mathematical models have been proposed, however criticism for them has been wide-ranging [20, 21]. For this reason the model introduced in this paper is built within the framework of a phenomenological approach in which blood coagulation is treated as a phase transition from a liquid to polymerized and gelled state [22–25]. To supplement the approach we introduce a set of closing relations that allow us to formally treat the polymerized and non-polymerized blood states in a unified manner.

We consider the problem in a two-dimensional approximation assuming a steady blood inflow for simplicity. The thrombogenic properties of the tissue surrounding the vessel are represented as a non-zero concentration of “primary activator” in the vicinity of the vessel wall. The activator can leak into the lumen through a permeable wall. Importantly, the permeability of the vessel is a function of shear stress on the vessel wall.

### 2.1 Hydrodynamics

**Fig 1:**
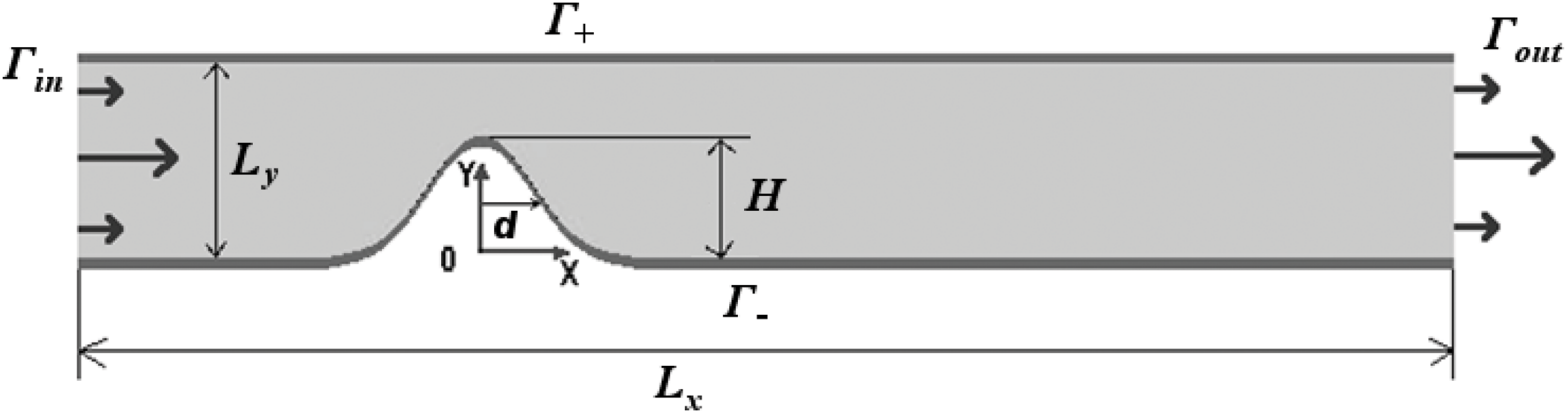
Geometry of the vessel fragment. *L*_*x*_, *L*_*y*_ and *H* correspond to vessel length, cross section diameter and plaque height. Γ_*in*_ and Γ_*out*_ denote inlet and outlet boundaries respectively. Γ_+_ and Γ_−_ refer to upper and lower vessel walls respectively.

The geometry of the problem is shown in Fig 1. The vessel walls are assumed to be rigid. For specificity, the vessel profile is approximated with the following formula:

$$f\left(x\right)=He^{-\frac{x^{2}}{2d^{2}}}\;x\in \left[-\left(1/3\right)L_{x};\left(2/3\right)L_{x}\right],$$ (1)

where *L*_*x*_ denotes vessel length, *H* denotes plaque height and *d* corresponds to one half of stenosis width (see Fig 1).

The blood flow is described using the modified Navier-Stokes equations [26]:

$$\frac{\partial \vec{V}}{\partial t}+(\vec{V},\vec{\nabla })\vec{V}=-\frac{1}{\rho }\vec{\nabla }\;p+\nu \nabla^{2}\vec{V}-\alpha_{p}\left(M_{1},M_{2}\right)\nu \vec{V}$$ (2)

$$(\vec{\nabla },\vec{V})=0,$$ (3)

where $\vec{V}$ is blood velocity, *t* denotes time, $\vec{\nabla }$ is the Hamilton’s operator, *p* is pressure, and *ρ* and *ν* depict blood density and kinematic viscosity, respectively.

The last term in Eq (2) aims to account for the effect of thrombus formation on the blood flow. The effect is described in terms of the Darcy’s filtration law, with the multiplier *α*_*p*_(*M*_1_, *M*_2_) standing for the filtration resistance of a blood clot (in case one has formed). The values of *M*_1_ and *M*_2_ reflect first and second statistical moments of fibrin polymer mixture (see section 2.3). An explicit dependence of *α*_*p*_ on *M*_1_ and *M*_2_ is given in section 2.4.

No-slip boundary conditions are assumed for the vessel walls Γ_+_ and Γ_−_. The inlet and outlet boundary conditions are given as follows:

$$V_{x}{|}_{\Gamma_{in}}=\frac{4V_{0}}{L_{y}^{2}}y\left(L_{y}-y\right)$$ (4)

$$V_{y}{|}_{\Gamma_{in}}=0$$ (5)

$${p|}_{\Gamma_{out}}=0,$$ (6)

where *L*_*y*_ denotes vessel width and *V*_0_ is inlet blood flow velocity in the center of the vessel.

### 2.2 Blood vessel and activation source

The pro-coagulant factors that enter the lumen from atherosclerotic plaques are mostly products of inflammatory processes that take place inside the plaques [27, 28]. In this work we do not focus on their biochemical nature and describe their effect in terms of a phenomenological “primary activator” of blood coagulation [22–25] denoted as *u*.

The concentration of primary activator under the vessel wall *u*_0_ is assumed to be non-zero in the vicinity of the stenosed wall Γ_−_. The “healthy” non-atherosclerotic tissue is assumed to be devoid of primary activator:

$$u_{0}^{+}=0$$ (7)

$$u_{0}^{-}=\{\begin{array}{ccc} & u_{0}, & x\in [-2d;2d]\\ & 0, & x\notin [-2d;2d]. \end{array}$$ (8)

The activator is assumed to be “leaking” into the vessel from the underlying tissue. In the mathematical model this is represented by the boundary condition imposed on *u* on Γ_±_:

$$-D_{u}\frac{\partial u}{\partial \vec{n}}{|}_{\Gamma_{\pm }}=\mu \left(\left|\tau \right|\right)\left(u_{0}^{\pm }-u{|}_{\Gamma_{\pm }}\right),$$ (9)

where *μ* is the vessel wall permeability, *D*_*u*_ denotes diffusion coefficient, operator ${\frac{\partial }{\partial \vec{n}}|}_{\Gamma_{\pm }}$ denotes the space derivative normal to Γ_±_, *u*∣_Γ_±__ is the concentration of the primary activator in the blood flow near the corresponding vessel wall (Γ_±_).

The permeability *μ* is assumed to depend on wall shear stress $\tau =\rho \nu \frac{\partial V}{\partial \vec{n}}$. The dependence is approximated with a piecewise-linear function:

$$\mu =\{\begin{array}{ccc} & \mu_{1}, & \left|\tau \right|\leq \tau_{1}\\ & \frac{\left|\tau \right|-\tau_{1}}{\tau_{2}-\tau_{1}}\left(\mu_{2}-\mu_{1}\right)+\mu_{1}, & \tau_{1}<\left|\tau \right|<\tau_{2}\\ & \mu_{2}, & \left|\tau \right|\geq \tau_{2}. \end{array},$$ (10)

where *τ*_1_ and *τ*_2_ are threshold shear stress values that define the degree of vessel wall resistance to stress; *μ*_1_ denotes the permeability of an intact vessel wall, while *μ*_2_ corresponds to the maximal permeability of the vessel.

The concentration of the primary activator *u* is taken to be zero on the inlet boundary Γ_*in*_. Zero-gradient conditions are assumed on Γ_*out*_ [29, 30].

### 2.3 Blood coagulation cascade

The kinetics of blood coagulation reactions is described in the framework of the phenomenological model introduced in [22–25]:

$$\frac{\partial u}{\partial t}=-k_{d}u-\nabla \cdot (\vec{V}u-D_{u}\nabla u)$$ (11)

$$\frac{\partial \theta }{\partial t}=k_{u}u+\frac{\alpha \theta^{2}}{\theta +\theta_{0}}-\chi_{1}\theta -\gamma \theta \varphi -\nabla \cdot (\vec{V}\theta -D_{\theta }\nabla \theta )$$ (12)

$$\frac{\partial \varphi }{\partial t}=\beta \theta \left(1-\frac{\varphi }{c}\right)\;\left(1+{\left(\frac{\varphi }{\varphi_{0}}\right)}^{2}\right)-\chi_{2}\varphi -\nabla \cdot \left(\vec{V}\varphi -D_{\varphi }\nabla \varphi \right)$$ (13)

$$\frac{\partial F_{g}}{\partial t}=-k_{g}F_{g}\theta -\epsilon_{g}(F_{g}-F_{g}^{0})-\nabla \cdot (\vec{V}F_{g}-D_{g}\nabla F_{g}).$$ (14)

Here *θ* and *φ* denote concentrations of the activator and the inhibitor of biochemical network of blood coagulation reactions (see [22, 31–33]) and *F*_*g*_ corresponds to fibrinogen (fibrin precursor) concentration. The components of the coagulation cascade are subject to convection and diffusion as described by the last term in Eqs (11)–(14).

The formation of fibrin polymers from fibrinogen is described in terms of the first two statistical moments of fibrin chain length distribution:

$$\frac{\partial M_{1}}{\partial t}=k_{g}F_{g}\theta -k_{r}M_{1}-\nabla \cdot (b_{p}\vec{V}M_{1}-D_{f}\nabla M_{1})$$ (15)

$$\frac{\partial M_{2}}{\partial t}=k_{g}F_{g}\theta +4k_{p}{\left(M_{2}+M_{1}\right)}^{2}-\frac{k_{b}}{3}(\frac{M_{2}^{2}}{M_{1}}-M_{1})-k_{r}M_{2}-\nabla \cdot (b_{p}\vec{V}M_{2}-D_{f}\nabla M_{2}),$$ (16)

The first and second fibrin moments *M*_1_ and *M*_2_ are defined through the concentration of *k*-mers of fibrin *F*_*k*_ as [22, 34]:

$$M_{n}=\sum_{k=1}^{\infty }k^{n}F_{k},\;\;n=1,\;2.$$ (17)

*M*_1_ reflects the total amount of fibrin-monomer molecules in polymerized and non-polymerized forms. The ratio *M*_2_/*M*_1_ determines weight-averaged number of fibrin-monomers in polymer molecules of fibrin [35–37]:

$$N_{w}=\frac{M_{w}}{m_{0}}=\frac{M_{2}}{M_{1}}.$$ (18)

Just as Eqs (11)–(14) the polymerization equations take into account diffusion and convection, accounting for the fact that, generally speaking, both depend on the polymer molecule size. This dependency is incorporated into the coefficient of polymer chains transport *b*_*p*_ and diffusion coefficient *D*_*f*_. The explicit expressions for *b*_*p*_ and *D*_*f*_ as functions of fibrin-polymer moments are given in the next section.

The values of *u*, *θ*, *φ*, *M*_1_ and *M*_2_ on the boundary Γ_*in*_ are supposed to be equal to zero in all numerical experiments, while *F*_*g*_ concentration on Γ_*in*_ is assumed to be equal to initial fibrinogen concentration $F_{g}^{0}$. The zero-gradient conditions were adopted for all chemicals on Γ_*out*_ [29, 30]. Vessel walls are taken to be impermeable for *θ*, *φ*, *F*_*g*_, *M*_1_ and *M*_2_.

### 2.4 Special features of polymers movement

To close the mathematical description explicit definition of *D*_*f*_, *b*_*p*_ and *α*_*p*_ is needed. For the purpose we develop a phenomenological approach that allows us to formally treat the polymerized and non-polymerized states of blood in a unified manner.

To describe dependence of polymer chains diffusion coefficient *D*_*f*_ on polymer chain size the following asymptotic expression is used:

$$D_{f}=D\cdot \frac{1}{N_{w}}\cdot \frac{1}{1+N_{w}/N_{e}},$$ (19)

where *N*_*e*_ is a phenomenological parameter of reptation theory [38, 39]. An asymptotic Eq (19) has very clear limit forms:

- when fibrin molecules experience only hydrodynamic friction [39, 40], *N*_*w*_ ≪ *N*_*e*_, for the diffusion coefficient one has: *D*_*f*_ ∼ 1/*N*_*w*_;
- in accordance with reptation theory [38, 39], when *N*_*w*_ ≫ *N*_*e*_ and $D_{f}∼1/N_{w}^{2}$.

The expression for *b*_*p*_ was obtained in the following way. Single polymer chain in fluid flow experiences the following drag force (we neglect hydrodynamic interaction effects in this work):

$${\vec{F}}_{flow}=\frac{kT}{D}\cdot N_{w}\cdot \vec{V}.$$ (20)

This force makes the chain to move with the speed:

$${\vec{V}}_{chain}=\frac{D_{f}}{kT}\cdot \frac{kT}{D}\cdot N_{w}\cdot \vec{V}=\frac{1}{1+N_{w}/N_{e}}\cdot \vec{V}.$$ (21)

Thus *b*_*p*_ is:

$$b_{p}=\frac{1}{1+N_{w}/N_{e}}.$$ (22)

It was assumed that in blood the prevailing mechanism of fibrin molecules nucleation is a heterogeneous one and that microparticles and some of the formal blood elements serve as nucleation centers [41]. If the concentration of heterogeneous centers of nucleation is equal to *n*_0_ ([*n*_0_] = [*cm* ^−3^]), the distance between the centers may be estimated as $n_{0}^{-1/3}$.

Assuming fibrin polymer chains to be Gaussian, characteristic size of the coil may be estimated as [40]:

$$R=l_{0}\sqrt{N_{w}K},$$ (23)

where *l*_0_ is the characteristic size of single fibrin-monomer molecule, and *K* is number of fibrin monomers in Kuhn’s segment. *l*_0_ can be estimated from the fibrin molecule volume: $l_{0}=\sqrt[{3}]{v_{0}}$.

The transition between diluted and semidiluted solution takes place when $N_{w}=N_{w}^{s}$ and when the distance between centers of nucleation is equal to *R*. This gives the value $N_{w}^{s}$[38]:

$$N_{w}^{s}=\frac{1}{n_{0}^{2/3}l_{0}^{2}K}.$$ (24)

It is easy to see that the coefficient of polymer chains transport by flow *b*_*p*_ is essentially decreased if *N*_*w*_ > *N*_*e*_. At the same time it is known that entanglement effects are essential when $N_{w}>N_{w}^{s}$. Hence, it seems to be reasonable to suppose that the magnitudes of *N*_*e*_ and $N_{w}^{s}$ are of the same value:

$$N_{e}\approx N_{w}^{s}.$$ (25)

Coefficient *α*_*p*_ determines the degree of slowing down the velocity of blood when large polymers or gel are formed. In this case *α*_*p*_ is a filtration resistance inversely proportional to filtration permeability in the Darcy’s law. In the case of dilute polymer solution there is almost no slowing effect, *α*_*p*_ is given by:

$$\alpha_{p}=\frac{1-b_{p}}{\xi^{2}},$$ (26)

where *ξ* represents characteristic size of polymer gel/solution. It is known that for diluted solutions *b*_*p*_ goes to unity. In accordance with expression (26) the value of filtration resistance drops to zero. The value of *ξ* reflects average pore size in gel and it is equal to the distance between polymer molecules in solution.

In dilute solution the distance between polymer molecules is more than molecules size *ξ* ≫ *R* and the average concentration of monomers in the solution, *M*_1_, is much smaller then the local concentration of monomers in one polymer *M*_*c*_: *M*_*c*_ ≫ *M*_1_. In mature gel opposite conditions are satisfied: *ξ* < *R*, *M*_*c*_ < *M*_1_. General form of expression for *ξ* may be given as [36, 38]:

$$\xi =R\cdot {\left(\frac{M_{c}}{M_{1}}\right)}^{m},$$ (27)

where power *m* must be chosen to make *ξ* independent on *N*_*w*_.

Local concentration of fibrin-monomer in the Gaussian coil is estimated as:

$$M_{c}=\frac{N_{w}}{N_{a}R^{3}},$$ (28)

where *N*_*w*_/*N*_*a*_ is a number of moles of monomers in one polymer (*N*_*a*_ is Avogadro number) and *R* ^3^ estimates the volume that polymer occupies.

Substituting Eq (28) to Eq (27) and choosing *m* = 1 [38] gives:

$$\xi =R\frac{N_{w}}{N_{a}R^{3}M_{1}}=\frac{N_{w}}{N_{a}R^{2}M_{1}}=\frac{1}{l_{0}^{2}KN_{a}M_{1}}.$$ (29)

This gives for *α*_*p*_:

$$\alpha_{p}=\alpha_{0}M_{1}^{2}\left(1-b_{p}\right),$$ (30)

where

$$\alpha_{0}=l_{0}^{4}K^{2}N_{a}^{2}.$$ (31)

Equations describing polymerization in terms of statistical moments may have singular solutions (particularly, when *M*_2_ blows up) [42]. In present work we focus on the early stages of fibrin gel formation in the blood flow. It was assumed that gel is worth to be considered as sufficiently “mature” when the mean weight-averaged number of fibrin-monomers in polymer molecules *N*_*w*_ exceeds the value corresponding to the semidiluted solution condition $N_{w}^{s}$ by at least two orders of magnitude ($N_{w}=10^{2}N_{w}^{s}$). Research of more condensed gel evolution remains outside the scope of this work’s objectives.

In the mathematical model presented in this work, if *N*_*w*_ exceeded “mature gel” value ($100\cdot N_{w}^{s}$) in some region in the vessel, then *M*_2_ at that region is assumed to be equal to its maximal value $100\cdot M_{1}\cdot N_{w}^{s}$.

### 2.5 Initial conditions and numerical parameter values

At the initial moment *t* = 0 all variables but *F*_*g*_ were assumed to be equal to zero in the interior part of calculation domain while *F*_*g*_ was assumed to be equal to $F_{g}^{0}$. The $\vec{V}$ and *p* fields are taken to correspond to stationary flow under given boundary conditions.

Numerical simulations were performed assuming *L*_*x*_ = 7.5 *cm* and *L*_*y*_ = 1 *cm*. The values of other parameters used in numerical calculations are listed in Table 1 (see also [24, 25]).

**Table 1:** Parameter values.

Parameter	Value	Refs.	Parameter	Value	Refs.
*α*	3.33 ⋅ 10^−2^ *s* ^−1^	[22, 23]	*k*_*b*_	1.67 ⋅ 10^−3^ *s* ^−1^	[22, 23]
*θ*_0_	5 *nM*	—“—	*n*_0_	10^10^ *cm* ^−3^	[43]
*χ*_1_	8.33 ⋅ 10^−4^ *s* ^−1^	—“—	$F_{g}^{0}$	9 ⋅ 10^3^ *nM*	[22, 23]
*γ*	8.33 ⋅ 10^−2^ (*nM* ⋅ *s*)^−1^	—“—	*D*_*u*_	3 ⋅ 10^−7^ *cm* ^2^/*s*	—“—
*β*	2.5 ⋅ 10^−5^ *s* ^−1^	—“—	*D*_*φ*_	3 ⋅ 10^−7^ *cm* ^2^/*s*	—“—
*c*	5 *nM*	—“—	*D*_*θ*_	3 ⋅ 10^−7^ *cm* ^2^/*s*	—“—
*ϵ*_*g*_	1.66 ⋅ 10^−6^ *s* ^−1^	—“—	*D*_*g*_	3 ⋅ 10^−7^ *cm* ^2^/*s*	—“—
*φ*_0_	0.05 *nM*	—“—	*D*	3 ⋅ 10^−7^ *cm* ^2^/*s*	—“—
*χ*_2_	0.35 *nM*	—“—	*k*_*d*_	1.66 ⋅ 10^−6^ *s* ^−1^	—“—
*k*_*g*_	5 ⋅ 10^−6^ (*nM* ⋅ *s*)^−1^	—“—	*k*_*u*_	1.66 ⋅ 10^1^ *s* ^−1^	—“—
*k*_*p*_	2.5 ⋅ 10^−4^ (*nM* ⋅ *s*)^−1^	—“—	*k*_*r*_	1.67 ⋅ 10^−2^ *s* ^−1^	—“—
*τ*_1_	10 *dyn*/*cm* ^2^	[15, 44, 45]	*μ*_1_	2 ⋅ 10^−12^ *cm*/*s*	[24, 25]
*τ*_2_	20 *dyn*/*cm* ^2^	[15, 44, 45]	*u*_0_	100 *nM*	[24, 25]
*ν*	5 ⋅ 10^−2^ *cm* ^2^/*s*	[43]	*ρ*	1 *g*/*cm* ^3^	[43]
*K*	10	[46]	*v*_0_	3 ⋅ 10^−18^ *cm* ^3^	[46, 47]

## 3 Numerical methods

Eqs (2)–(14), (15) and (16) were solved numerically by means of finite volume method [48, 49] with the aid of splitting technique [30, 50]. To discretize convective terms in Eqs (11)–(14), (15) and (16) upwind differencing was used [30, 48]. The Laplacian term was discretized by the means of “over-relaxed correction” technique [49]. To solve equations of fluid motion Eqs (2) and (3) PISO method was used [49, 51] with linear approximation of convective terms. Chemical kinetics ODE’s were solved by backward differencing technique [30, 52].

The program code was written with the help of OpenFOAM [53] and SUNDIALS [52] open-source libraries. Numerical simulations were carried out on unstructured meshes consisting of both triangles and quadrangles. The boundary layers in the vicinity of the vessel walls were meshed by quadrangles whereas the other parts of mesh were meshed by triangles. The meshes were generated using the open-source program SALOME [54] and adaptively refined in the near-wall area and along the separatrix line (see section 4.3). The results were visualized using ParaView [55] and gnuplot [56] open-source programs.

## 4 Results

Eqs (2), (3) and (11)–(16) with boundary conditions (1)–(6) and (9) and initial conditions described in section 2.5 were solved numerically. Generally speaking, the system considered is governed by two dozens of dimensionless “true” parameters easily determined by scaling procedures [57]. We have focused our analysis on the dependence of the solution on following four non-dimensional parameters related to blood flow intensity, wall permeability and plaque shape:

$$Re=\frac{V_{0}L_{y}}{\nu }$$ (32)

$${\tilde{\mu }}_{2}=\mu_{2}u_{0}\cdot \frac{k_{u}}{L_{y}{\left(\alpha -\chi_{1}\right)}^{2}\theta_{0}}$$ (33)

$$\tilde{d}=d/L_{y}$$ (34)

$$h=H/L_{y}.$$ (35)

The rest of the parameters were held fixed as in Table 1.

### 4.1 Threshold-like activation of intravascular thrombus formation

Of primary importance is mapping the blood coagulation regimes in the system. We define coagulation in terms of fibrin gelation occurring anywhere in the vessel. Mathematically, this corresponds to *N*_*w*_ = *N*_*w*_(*x*, *y*, *t*) reaching $N_{w}^{s}$ in the flow domain at a finite time. In contrast, the no thrombi formation regime is defined such that $N_{w}<N_{w}^{s}$ holds true everywhere in the vessel.

**Fig 2:**
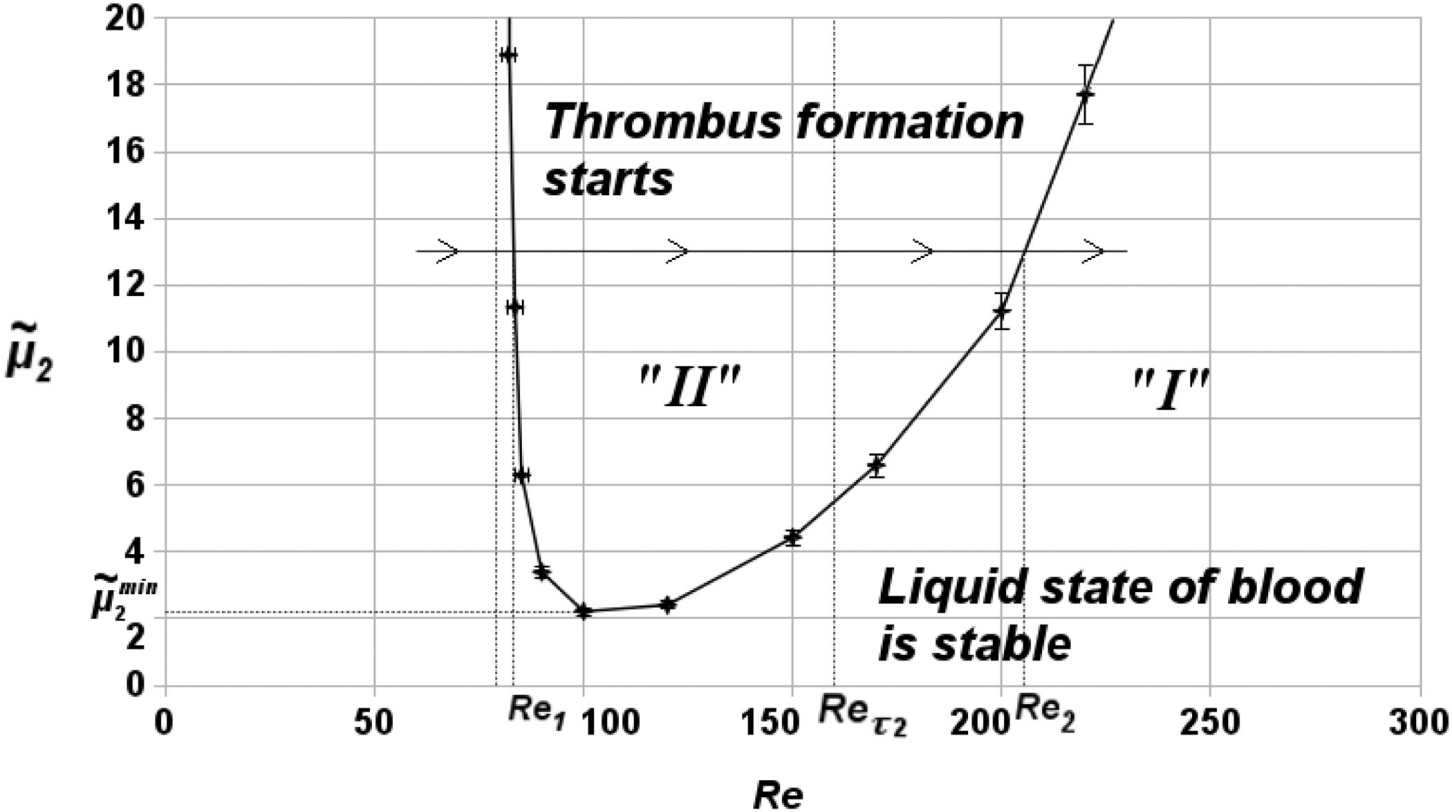
Parametric diagram of blood coagulation system regimes in the $\left(Re,{\tilde{\mu }}_{2}\right)$ parameter space. *Re* is the Reynolds number and ${\tilde{\mu }}_{2}$ is the non-dimensional vessel wall permeability. Label “I” is used to denote stationary regimes with no coagulation in the vessel while “II” marks the regimes with thrombi formation. Also shown are two typical values *Re*_1_, *Re*_2_ marking the boundary of the coagulation regime for a given ${\tilde{\mu }}_{2}$ and ${\tilde{\mu }}_{min}$—the permeability of the vessel wall below which clotting does not occur under any hydrodynamic conditions. Finally, *Re* = *Re*_*τ*2_ indicates the lowest Reynolds number at which the shear stress reaches *τ*_2_ and the local permeability of the vessel reaches $\tilde{\mu_{2}}$. *h* = 0.6, *d* = 0.4.

Numerical analysis of Eqs (2), (3) and (11)–(16) shows that both regimes — with and without blood coagulation — are observed in the system within the explored range of parameters. Fig 2 shows the parametric diagram of the regimes in the $\left(Re,{\tilde{\mu }}_{2}\right)$ parameter space. Label “I” is used to denote the stationary regimes with no coagulation in the vessel while “II” marks the regimes with thrombi formation.

Note the characteristic “tongue” shape of the boundary between regions I and II. This shape implies that the range of Reynolds number values at which blood coagulation occurs is limited both from above and below: for any ${\tilde{\mu }}_{2}>{\tilde{\mu }}_{2}^{min}$ two thresholds of hydrodynamic activation of blood coagulation exist. (${\tilde{\mu }}_{2}^{min}$ denotes the value of vessel wall permeability such that if ${\tilde{\mu }}_{2}<{\tilde{\mu }}_{2}^{min}$ clotting does not occur under any hydrodynamic conditions (see Fig 2).)

The two thresholds can be naively interpreted as follows. Let us denote the two threshold *Re* values, for a given ${\tilde{\mu }}_{2}$, as *Re*_1_, *Re*_2_ (see Fig 2). If *Re* < *Re*_1_, the value of wall shear stress is small and primary activator *u* does not leak into the blood flow in sufficient quantities to induce coagulation. If *Re* > *Re*_2_, convective flow washes the coagulation substances away. If *Re*_1_ < *Re* < *Re*_2_, thrombus formation starts.

Note that the results imply that blood coagulation can be induced in the system via both increasing and decreasing the blood flow intensity — depending on the initial state of the system.

In addition to mapping the boundary between the two coagulation regimes we have explored the scaling behavior of the system in the vicinity of the boundary. Numerical simulations show that the following holds true for the nucleation time T* (given constant *Re*):

$$\left({\tilde{\mu }}_{2}-{\tilde{\mu }}_{2}^{crit}\right)T^{*\;3}=C_{1}=const.$$ (36)

Here the nucleation time T* is the non-dimensional time it takes for *N*_*w*_ to reach $N_{w}^{s}$ in least one point of the vessel in the simulation, ${\tilde{\mu }}_{2}^{crit}$ is the specific value of ${\tilde{\mu }}_{2}$, located on the border between zones “I” and “II”, and *C*_1_ is a value independent of ${\tilde{\mu }}_{2}$. Also, in the vicinity of the left portion of the boundary the following scaling law is valid (given constant ${\tilde{\mu }}_{2}$):

$$\left(Re-Re_{crit}\right)T^{*\;3}=C_{2}=const,$$ (37)

where *Re*_*crit*_ is the specific value of *Re*, located on the border between zones “I” and “II”, and *C*_2_ is the value independent on Reynolds number (*Re*).

In other words, it appears that in the vicinity of the liquid state stability boundary the clot nucleation time grows up to infinity (see Eqs (36) and (37)). This result is reminiscent of the results of the theory of first-order transitions where similar scaling laws exist connecting the extent of supersaturation with the nucleation time [41, 58, 59].

### 4.2 Effects of stenosis geometry

**Fig 3:**
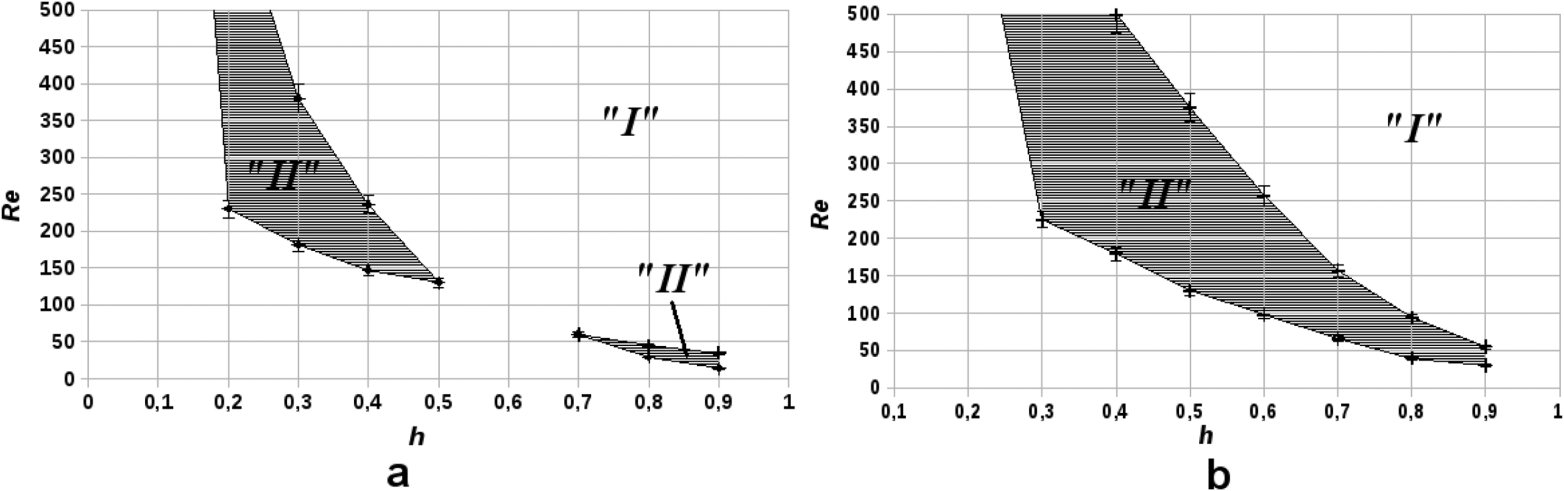
Parametric diagrams of blood coagulation regimes in the (*h*, *Re*) parameter space for two different values of $\tilde{d}$, the non-dimensional plaque diameter. Region “I” denotes regimes in which there is no coagulation, region “II” represents regimes of macroscopic thrombus formation. (a): $\tilde{d}=0.3$, ${\tilde{\mu }}_{2}=7$; (b): $\tilde{d}=0.5$, ${\tilde{\mu }}_{2}=7$.

We further investigated the coagulation regimes by exploring the influence of stenosis geometry on thrombus formation. Fig 3 shows the parametric diagrams in the (*h*, *Re*) space for two values of plaque width $\tilde{d}$. As in Fig 2 zone “I” corresponds to stable liquid states (no coagulation) and zone “II” corresponds to thrombus formation.

The results suggest that for relatively wide stenoses ($\tilde{d}=0.5$, Fig 3b) the “thrombogenic” zone “II” in parametric plane is a single-connected domain, while for a narrow stenosis ($\tilde{d}=0.3$, Fig 3a) zone “II” is split into two separate areas. There exists a critical value of $\tilde{d}$ for which zone “II” consist of two areas connected in 1 point only (not shown in Fig 3). This means that in parametric space $\left(h,\tilde{d},Re\right)$ the surface dividing zones “I” and “II” is saddle-like.

Interestingly, the results of the mapping also suggest that the range of Reynolds numbers in which macroscopic thrombus formation occurs in general is wider for relatively small plaques (0.3 < *h* < 0.4) than it is for large plaques (0.8 < *h* < 0.9), irrespective of $\tilde{d}$.

### 4.3 Typical scenarios

We also explored the spatial characteristics of blood coagulation in the system under consideration. Numerical simulations suggest that at least two different types of scenarios can be distinguished.

**Fig 4:**
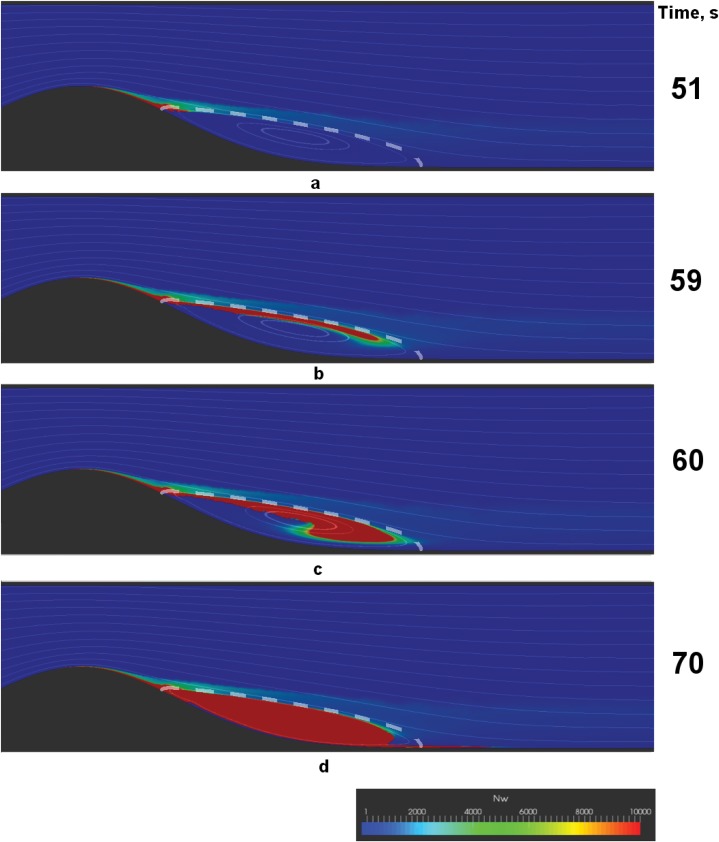
Successive stages of a solid thrombus formation. a—thrombus nucleation, b—formation of fibre-like fibrin structure, c—fibre-like structure thickening, d—solid thrombus. Shown are the color maps of *N*_*w*_—the weight-average number of monomers in fibrin-polymer in the vessel, with red areas representing regions of fibrin gel formation ($N_{w}\geq N_{w}^{s}$). Streamlines are plotted to visualize the flow, and the separatrix, which divides the core of the flow from the recirculation zone, is shown with a dashed line. Parameters used in the simulations are: *Re* = 130, *h* = 0.5, $\tilde{d}=0.5$, ${\tilde{\mu }}_{2}=9.5$. Note that only a fragment of the vessel closest to the plaque is depicted.

**Fig 5:**
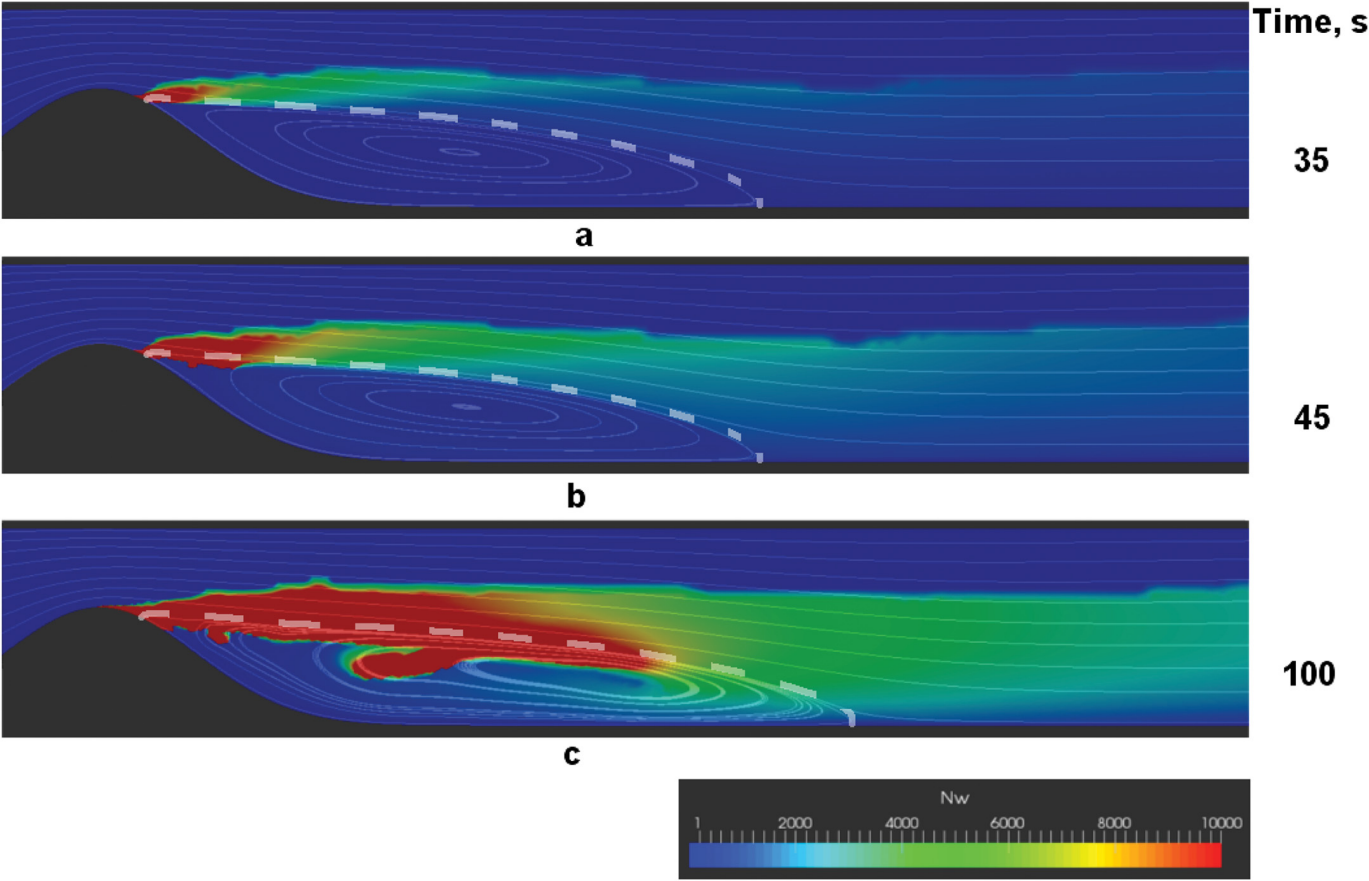
Successive stages of a friable floating fibrin structure formation. a—thrombus nucleation, b—formation of fibre-like fibrin structure, c—thick and friable floating fibrin structure. Shown are the color maps of *N*_*w*_—the weight-average number of monomers in fibrin-polymer in the vessel, with red areas representing regions of fibrin gel formation ($N_{w}\geq N_{w}^{s}$). Streamlines are plotted to visualize the flow, and the separatrix is shown with a dashed line. Parameters used in these simulations are: *Re* = 180, *h* = 0.6, $\tilde{d}=0.4$, $\tilde{d}=12$. Note that only a fragment of the vessel closest to the plaque is depicted.

Successive stages of the two different scenarios are shown in Figs 4 and 5. The figures show the distribution of the weight-average number of fibrin monomers in polymer chains *N*_*w*_ in the vessel. Red color corresponds to clots ($N_{w}\geq N_{w}^{s}$), while green shades represent microthrombi with different lengths of polymer chains ($N_{w}<N_{w}^{s}$).

Notably, the early stages of coagulation are the same across the explored range of parameters: the nucleation of a macroscopic thrombus occurs near the reattachment point of the recirculation zone that forms behind the stenosis. Then the stage of macroscopic fibre-like structure formation comes (see Figs 4b and 5b). The direction of the growth of the fibre-like structure is determined by the separatrix line, which divides the core of the flow from the recirculation zone.

Further behavior depends on the parameter values. The fibre either successively thickens into a solid thrombus in the recirculation zone (see Fig 4d) or underlays the formation of a floating friable structure (see Fig 5c). Interestingly, the floating structure does not have a sharp border and is characterized by a downstream cloud of mircothrombi.

## 5 Discussion

In this paper we develop a mathematical model to describe the clinically important case of thrombus formation in the vicinity of an atherosclerotic plaque. The clotting is induced by pro-coagulants leaking into the vessel from the sclerotic tissue through the permeable vessel wall. In contrast to earlier work in the field (see, e.g. [11, 60–67]) the dependence of the vessel wall permeability on shear stress is taken into account. The model sheds some light on the role shear stress plays in thrombogenicity of the vessel when the shear rates are too small to induce platelet activation, as soon as in the present work the inequation $\tau /(\rho \nu )=\dot{\gamma }\leq 10^{3}\;s^{-1}$ was valid at all times.

Within the scope of the model blood coagulation is treated as a phase transition from a liquid to polymerized and gelled state. To simplify the analysis across the different states we develop a phenomenological approach that allows us to formally treat the polymerized and non-polymerized blood in a unified manner. This is done through the closing expressions (19), (22) and (30) for *D*_*f*_, *b*_*p*_ and *α*_*p*_, implying that these coefficients can be described as functions of statistical moments of fibrin polymer distribution *M*_1_ and *M*_2_. The expressions (19), (22) and (30) are based on the scaling approach [38] and the perturbation theory technique of asymptotic expansions [68].

Numerical analysis of the introduced phenomenological model reveals the existence of two types of thresholds in hydrodynamic activation of blood coagulation (see Fig 2). One is associated with the increase of wall permeability to procoagulants above the critical level due to increased shear stresses in the vicinity of stenosis. The second threshold is concerned with the intensity with which the procoagulants are washed out from the atherosclerotic region.

The model also sheds some light onto the link between the risk of atherothrombosis and the associated stenosis’ shape and severity. Interestingly, plaques with severe stenosis (*h* > 0.6) induced clotting only at relatively low Reynolds number (*Re* < 200), while smaller plaques are characterized by a wider range of Reynolds numbers at which macroscopic thrombus formation occurs (Fig 3).

The result suggests that the most thrombogenic plaques are not necessarily those associated with the most severe occlusion and that the worldwide-accepted indications for stenting vessels should be critically re-assessed [69–71].

Finally, we describe two possible scenarios of blood coagulation in the vicinity of an atherosclerotic plaque. The first scenario corresponds to localized thrombus formation in the recirculation zone behind the stenosis (see Fig 4). The second scenario corresponds to the formation of a friable fibrin polymer structure flattering in the flow behind the plaque (see Fig 5). Which of these scenarios is realized in the system depends upon the blood flow conditions. Low Reynolds numbers usually correspond to solid thrombus formation. In contrast, intensive flows result in the formation of a flattering clot.

Importantly, the friable floating clots are characterized by loose long “tails” of microthrombi downstream of the stenosis (see Fig 5c). One can argue that this microthrombi “dust” may lead to microcirculatory disorders in organs distal from the plaque.

It is worth mentioning that in the case of loose thrombus formation, fibrin generation proceeds inside the thrombus. Thrombin generation continues inside the clot structure. It is natural to suppose that the more intense is thrombin generation the denser microthrombi clouds are formed downstream. Thereupon, an old conclusion may be repeated: to estimate thrombogenic perspectives there is more important to asses endogenous thrombin generation potential than time of clot formation (traditional measured by prothrombin or APTT tests) [72]. Endogenous thrombin potential controls the capability of blood for intensive generation of fibrin microthrombi (irrelative of whether they will be condensed on macrothrombus or washed out downstream) [73].

The floating fibre-like thrombi attached to stenosis are routinely observed in our in vitro arrangement for studying blood coagulation [74, 75]. Interestingly, these frail clots should flatter in the pulsating blood flow and thus be acoustically detectable. Presumably, in the past medical doctors could diagnose them by stethoscoping the sclerosed vessels.

It should be noted that the mathematical description adopted in this work has a few important limitations. In particular, we confine our analysis to stationary flows and neglect the dependence of blood viscosity upon fibrin polymerization. We hope to address these limitations in our future work. Yet, we believe that the results described in this paper will serve as a valuable guide for subsequent work in studying atherothrombosis.

## References

[1] Badimon L, Storey RF, Vilahur G. Update on lipids, inflammation and atherothrombosis. Thrombosis and Haemostasis. 2011;105 (Suppl 1):S34–S42. 10.1160/THS10-11-0717 21479344

[2] Davies MJ. The pathophysiology of acute coronary syndromes. Heart. 2000;83:361–366. 10.1136/heart.83.3.361 10677422 PMC1729334

[3] Makris GC, Nicolaides AN, Xu XY, Geroulakos G. Introduction to the biomechanics of carotid plaque pathogenesis and rupture: review of the clinical evidence. The British Journal of Radiology. 2010;83:729–735. 10.1259/bjr/49957752 20647514 PMC3473420

[4] Melkumyants AM. Role of Endothelial Glycocalyx in Mechanogenic Control of Arterial Hydraulic Resistance. Uspekhi fiziologicheskikh nauk. 2012;43(4):45–58. In Russian. 23227721

[5] Sadat U, Teng Z, Gillard JH. Biomechanical structural stresses of atherosclerotic plaques. Expert Rev Cardiovasc Ther. 2010;8(10):1469–1481. 10.1586/erc.10.130 20936933

[6] Shirinskii VP. The role of light-chain myosin kinase in endothelial barrier functions and the prospects for use of its inhibitors in impaired vascular permeability. Cardiologichesky vestnik. 2006;1(XIII):39–42.

[7] Shirinsky VP. Molecular Physiology of Endothelium and Mechanisms of Vascular Permeability. Uspekhi fiziologicheskikh nauk. 2011;42(1):18–32. In Russian. 21442955

[8] van Hinsbergh VWM. Endothelium—role in regulation of coagulation and inflammation. Semin Immunopathol. 2012;34:93–106. 10.1007/s00281-011-0285-5 21845431 PMC3233666

[9] Virchow R. Phlebose und Thrombose im Gefabsystem. Frankfurt: Gesammelte Abhandlungen zur wissenschaftlichen Medizin; 1856.

[10] Reininger AJ, Heinzmann U, Reininger CB, Friedrich P, Wurzinger LJ. Flow mediated fibrin thrombus formation in an endothelium-lined model of arterial branching. Thrombosis Research. 1994;74(6):629–641. 10.1016/0049-3848(94)90219-4 8091405

[11] Runyon MK, Kastrup CJ, Johnson-Kerner BL, Thuong G, Ha V, Ismagilov RF. Effects of Shear Rate on Propagation of Blood Clotting Determined Using Microfluidics and Numerical Simulations. JACS. 2008;130(11):3458–3464. 10.1021/ja076301r 18302373

[12] Lee KW, Lip GYH. Acute coronary syndromes: Virchow’s triad revisited. Blood Coagulation and Fibrinolysis. 2003;14:605–625. 10.1097/00001721-200310000-00001 14517485

[13] Shah PK. Inflammation and Plaque Vulnerability. Cardiovasc Drugs Ther. 2009;23:31–40. 10.1007/s10557-008-6147-2 18949542

[14] Wu KK, Thiagarajan P. Role of endothelium in thrombosis and hemostasis. Annu Rev Med. 1996;47:315–331. 10.1146/annurev.med.47.1.315 8712785

[15] Slager CJ, Wentzel JJ, Gijsen FJH, Thury A, van der Wal AC, Schaar JA, et al The role of shear stress in the destabilization of vulnerable plaques and related therapeutic implications. Nat Clin Pract Cardiovasc Med. 2005;2(9):456–464. 10.1038/ncpcardio0298 16265586

[16] Dirksen MT, van der Wal AC, van den Berg FM, van der Loos CM, Becker AE. Distribution of Inflammatory Cells in Atherosclerotic Plaques Relates to the Direction of Flow. Circulation. 1998;98:2000–2003. 10.1161/01.CIR.98.19.2000 9808596

[17] Alevriadou BR, Moake JL, Turner NA, Ruggeri ZM, Folie BJ, Phillips MD, et al Real-time analysis of shear-dependent thrombus formation and its blockade by inhibitors of von Willebrand factor binding to platelets. Blood. 1993;81(5):1263–1276. 8443388

[18] Ruggeri ZM. Mechanisms of shear-induced platelet adhesion and aggregation. Thromb Haemost. 1993;70(1):119–123. 8236086

[19] Ruggeri ZM, Orje JN, Habermann R, Federici AB, Reininger AJ. Activation-independent platelet adhesion and aggregation under elevated shear stress. Blood. 2006;108:1903–1910. 10.1182/blood-2006-04-011551 16772609 PMC1895550

[20] Hemker HC, Kerdelo S, Kremers RMW. Is there value in kinetic modeling of thrombin generation? No (unless…). Journal of Thrombosis and Haemostasis. 2012;10:1470–1477. 10.1111/j.1538-7836.2012.04802.x 22650179

[21] Wagenvoord R, Hemker PW, Hemker HC. The limits of simulation of the clotting system. Journal of Thrombosis and Haemostasis. 2006;4(6):1331–1338. 10.1111/j.1538-7836.2006.01967.x 16706979

[22] Guria GT, Herrero MA, Zlobina KE. A mathematical model of blood coagulation induced by activation sources. Discr Cont Dyn Syst A. 2009;25(1):175–194. 10.3934/dcds.2009.25.175

[23] Guria GT, Herrero MA, Zlobina KE. Ultrasound detection of externally induced microthrombi cloud formation: a theoretical study. Journal of Engineering Mathematics. 2010;66(1–3):293–310. 10.1007/s10665-009-9340-9

[24] Rukhlenko AS, Dudchenko OA, Zlobina KE, Guria GT. Threshold activation of blood coagulation as a result of elevated wall shear stress. Proceedings of MIPT. 2012;4(2):192–201. In Russian.

[25] Rukhlenko AS, Zlobina KE, Guria GT. Hydrodynamical activation of blood coagulation in stenosed vessels. Theoretical analysis. Computer Research and Modeling. 2012;4(1):155–183. In Russian.

[26] Nield DA, Bejan A. Convection in Porous Media. vol. XXIV 3rd ed Springer; 2006.

[27] Davies MT. Stability and instability two faces of coronary atherosclerosis. Circulation. 1994;90:2013–2019. 10.1161/01.cir.94.8.2013 8873680

[28] Ross R. Atherosclerosis—an inflammatory disease. N Engl J Med. 1999;340:115–126. 10.1056/NEJM199901143400207 9887164

[29] Hirsch C. Numerical computation of internal and external flows: fundamentals of computational fluid dynamics. vol. 1 2nd ed Elsevier/Butterworth-Heinemann; 2007.

[30] Oran ES, Boris JP. Numerical Simulation of Reactive Flow. Elsevier; 1987.

[31] Ataullakhanov FI, Guria GT. Spatial aspects of human blood clotting dynamics I. Hypothesis. Biophysics. 1994;39(1):89–96. 8161593

[32] Ataullakhanov FI, Guria GT, Safroshkina AY. Spatial aspects of human blood clotting dynamics II. Phenomenological model. Biophysics. 1994;39(1):97–104. 8161594

[33] Guria K, Guria GT. Spatial aspects of blood coagulation: Two decades of research on the self-sustained traveling wave of thrombin. Thrombosis Research. 2015;135(3):423–433. 10.1016/j.thromres.2014.12.014 25550187

[34] Echeverri LF, Herrero MA, Lopez JM, Oleaga G. Early Stages of Bone Fracture Healing: Formation of a Fibrin–Collagen Scaffold in the Fracture Hematoma. Bull Math Biol. 2015;77(1):156–183. 10.1007/s11538-014-0055-3 25537828

[35] Friedlander SK. Smoke, Dust, and Haze: Fundamentals of Aerosol Dynamics. Oxford; 2000.

[36] Strobl G. The Physics of Polymers. Concepts for Understanding Their Structures and Behavior. 3rd ed Springer-Verlag Berlin Heidelberg; 2007.

[37] Volkenstein MV. Molecular biophysics. New York: Academic press; 1977.

[38] de Gennes PG. Scaling Concepts in Polymer Physics. Cornell University Press; 1979.

[39] Doi M, Edwards SF. The Theory of Polymer Dynamics. Oxford University Press; 1988.

[40] Grosberg AY, Khokhlov AR. Statistical Physics of Macromolecules. AIP Press; 1994.

[41] Schmelzer JWP, editor. Nucleation Theory and Applications. Wiley; 2005.

[42] Herrero MA. Mathematical models of aggregation: the role of explicit solutions. Progress in nonlinear differential equations and their applications. 2005;63:309–318. 10.1007/3-7643-7384-9_31

[43] Schmidt RF, Thews G. Human Physiology. New York: Springer-Verlag; 1989.

[44] Tarbell JM. Shear stress and the endothelial transport barrier. Cardiovascular Research. 2010 7 15;87(2):320–330. 10.1093/cvr/cvq146 20543206 PMC2915475

[45] Gertz SD, Roberts WC. Hemodynamic Shear Force in Rupture of Coronary Arterial Atherosclerotic Plaques. The American Journal Of Cardiology. 1990;66:1368–1372. 10.1016/0002-9149(90)91170-B 2244569

[46] Weisel JW. Fibrinogen and Fibrin In: Parry DAD, Squire JM, editors. Fibrous Proteins: Coiled-Coils, Collagen and Elastomers. vol. 70 of Advances in Protein Chemistry. Academic Press; 2005 p. 247–299. 10.1016/S0065-3233(05)70008-5 15837518

[47] Bachmann L, Schmittfumian WW, Hammel R, Lederer K. Size and shape of fibrinogen. 1. Electron-microscopy of hydrated molecule. Makromol Chem-Macromol Chem Phys. 1975;176(9):2603–2618. 10.1002/macp.1975.021760912

[48] Patankar SV. Numerical Heat Transfer and Fluid Flow. Taylor & Francis; 1980.

[49] Jasak H. Error analysis and estimation for the Finite Volume method with applications to fluid flows [PhD Thesis]. Imperial College. London; 1996.

[50] Lobanov AI, Starozhilova TK, Guriya GT. Numerical investigation of pattern formation in blood coagulation. Mathematical modeling. 1997;9:83–95. In Russian.

[51] Issa RI. Solution of the implicitly discretized fluid flow equations by operator-splitting. J Comp Physics. 1986;62:40–65. 10.1016/0021-9991(86)90099-9

[52] Hindmarsh AC, Brown PN, Grant KE, Lee SL, Serban R, Shumaker DE, et al SUNDIALS: Suite of Nonlinear and Differential/Algebraic Equation Solvers. ACM Transactions on Mathematical Software. 2005;3(31):363–396. 10.1145/1089014.1089020

[53] OpenFOAM. The Open Source CFD Toolbox User Guide. OpenCFD Limited; 2009.

[54] http://www.salome-platform.org/.

[55] Henderson A. ParaView Guide, A Parallel Visualization Application. Kitware Inc; 2007.

[56] http://www.gnuplot.info/.

[57] Barenblatt GI. Scaling. Cambridge University Press; 2003.

[58] Lifshitz IM, Slyozov VV. The kinetics of precipitation from supersaturated solid solutions. J Phys Chem Solids. 1961;19:35–50. 10.1016/0022-3697(61)90054-3

[59] Slezov VV. Kinetics of First-Order Phase Transitions. John Wiley & Sons; 2009.

[60] Guy RD, Fogelson AL, Keener JP. Fibrin gel formation in a shear flow. Math Med Biol. 2007;24(1):111–130. 10.1093/imammb/dql022 17018571

[61] Guzevatykh AP, Lobanov AI, Guria GT. Threshold intravascular blood coagulation as a result of stenosis development. Mathematical modeling. 2000;12(4):39–60. In Russian.

[62] Chulichkov AL, Nikolaev AV, Lobanov AI, Guria GT. Threshold activation of blood coagulation and thrombus growth under flow conditions. Mathematical modeling. 2000;12(3):76–95. In Russian.

[63] Anand M, Rajagopal K, Rajagopal KR. A model incorporating some of the mechanical and biochemical factors underlying clot formation and dissolution in flowing blood. J of Theoretical Medicine. 2003;5:183–218. 10.1080/10273660412331317415

[64] Anand M, Rajagopal K, Rajagopal KR. A model for the formation and lysis of blood clots. Pathophysiol Haemost Thromb. 2005;34:109–120. 10.1159/000089931 16432312

[65] Lobanov AI, Starozhilova TK. Effect of convective flow on formation of two-dimensional structures in the model of blood coagulation. Phystech Journal. 1997;3(2):96–105.

[66] Lobanov AI, Starozhilova TK. The Effect of Convective Flows on Blood Coagulation Processes. Pathophysiol Haemost Thromb. 2005;34:121–134. 10.1159/000089932 16432313

[67] Neeves KB, Illing DAR, Diamond SL. Thrombin flux and wall shear rate regulate fibrin fiber deposition state during polymerization under flow. Biophysical Journal. 2010;98(7):1344–1352. 10.1016/j.bpj.2009.12.4275 20371335 PMC2849060

[68] Nayfeh AH. Perturbation Methods Physics textbook. Wiley; 2008.

[69] Eeckhout E, et al Indications for intracoronary stent placement: the European view. European Heart Journal. 1999;20(14):1014–1019. 10.1053/euhj.1998.1395 10381853

[70] Bates, et al ACCF/SCAI/SVMB/SIR/ASITN 2007 Clinical Expert Consensus Document on Carotid Stenting. Journal of the American College of Cardiology. 2007;49(1):126–170. 10.1016/j.jacc.2006.10.021 17207736

[71] Patel, et al ACCF/SCAI/STS/AATS/AHA/ASNC/HFSA/SCCT 2012 Appropriate Use Criteria for Coronary Revascularization Focused Update. Journal of the American College of Cardiology. 2012;59(9):857–881. 10.1016/j.jacc.2011.12.001 22296741

[72] Favaloro EJ, Lippi G. Coagulation update: What’s new in hemostasis testing? Thrombosis Research. 2011;127(Suppl. 2):S13–S16. 21193107 10.1016/S0049-3848(10)70148-1

[73] Dieri RA, de Laat B, Hemker HC. Thrombin generation: What have we learned? Blood Reviews. 2012;26:197–203. 22762893 10.1016/j.blre.2012.06.001

[74] Uzlova SG, Guria KG, Shevelev AA, Vasiliev SA, Guria GT. Acoustically detectable intravascular microemboli as precursors of postoperative complications. Bulletin of Bakoulev Scientific Center for Cardiovascular Surgery. 2008;(6):55–64. In Russian.

[75] Uzlova SG. Acoustical detection of microemboli on early stages *in vitro* and *in vivo* [PhD Thesis]. National Research Center for Hematology. Moscow; 2009.

